# Influence of Horizontal Model Resolution on the Horizontal Scale of Extreme Precipitation Events

**DOI:** 10.1029/2023JD040146

**Published:** 2024-11-23

**Authors:** S. M. Anas Ali, Neil F. Tandon

**Affiliations:** ^1^ Department of Earth and Space Science and Engineering York University Toronto ON Canada; ^2^ Department of Physics University of Toronto Toronto ON Canada

**Keywords:** extreme precipitation, global climate models, model resolution, atmospheric dynamics, convective parameterization

## Abstract

A fundamental characteristic of extreme precipitation events (EPEs) is their horizontal scale. This horizontal scale can influence the intensity of an EPE through its effect on the timescale of an EPE as well as its effect on the strength of convective feedbacks. Thus, to have confidence in future projections of extreme precipitation, the horizontal scales of EPEs in global climate models (GCMs) should be evaluated. Analyzing daily output from 27 models participating in the Coupled Model Intercomparison Project phase 6 (CMIP6), including 13 models participating in the High Resolution Model Intercomparison Project (HighResMIP), we computed the horizontal scales of EPEs and extreme ascent for annual maximum EPEs during 1981–2000. We found that the horizontal scales of both EPEs and the associated ascending motion are resolution‐dependent: for a factor of seven increase in horizontal resolution, the horizontal scale decreases by a factor of approximately two to five, with higher sensitivity in the tropics than in the midlatitudes. Further analysis in the southern hemisphere midlatitudes reveals that this resolution dependence results from precipitation during the simulated EPEs that is almost entirely resolved rather than parameterized. However, the EPEs are not simply grid box storms, and analysis of the horizontal scales of geopotential anomalies suggests that the planetary‐scale dynamics in GCMs is not resolution‐dependent. Thus, the dominance of resolved precipitation during EPEs is more likely due to convection on the model grid or formation of strong, poorly resolved fronts, and additional work is needed to explore these possibilities and find a remedy for this resolution dependence.

## Key Points

- The simulated horizontal scale of the annual maximum of daily precipitation anomalies is sensitive to a model's horizontal resolution
- This extreme precipitation horizontal scale is determined almost entirely by resolved, not parameterized, precipitation
- The simulated horizontal scale of geopotential disturbances during these extreme precipitation events is not resolution dependent

## 1 Introduction

Under climate change, extreme precipitation events (EPEs) are projected to intensify over most of the globe due to the thermodynamic increase in specific humidity following the Clausius‐Clapeyron relationship (e.g., Kharin et al., 2013; O’Gorman & Schneider, 2009). Studies examining EPE projections from global climate models (GCMs) typically focus on the maximum of daily precipitation for a given return period or high percentile (e.g., Kharin et al., 2013; O’Gorman & Schneider, 2009; Pfahl et al., 2017; Tandon et al., 2018). The projected intensification of EPEs means that for a given EPE, the amount of precipitation—i.e. the extreme precipitation intensity (EPI)—is expected to increase. However, this thermodynamic effect does not capture all of the mechanisms driving extreme precipitation changes. This is because dynamical effects associated with changes in extreme ascent (the upward vertical velocity during an EPE) strongly influence EPI in particular regions (Pfahl et al., 2017; Tandon et al., 2018). Most GCMs project that, with warming, there will be increased extreme ascent in the deep tropics, thus amplifying thermodynamic EPI increases, and decreased extreme ascent in the subtropical dry zones, thus opposing thermodynamic EPI increases (Pfahl et al., 2017).

These extreme ascent changes are the greatest source of uncertainty in extreme precipitation projections (Pfahl et al., 2017), but the reasons behind these changes remain poorly understood. Recent work has analyzed several potential drivers of extreme ascent changes. Pendergrass (2020) has proposed that changes in convective organization might drive future increases in extreme ascent in the tropics. As for projected extreme ascent decreases in the subtropics, Pfahl et al. (2017) and Norris et al. (2020) have suggested that expansion of the Hadley cells may be partly responsible, but Tandon et al. (2018) have argued that these changes likely result from increases in the horizontal scale of ascent. Furthermore, Li and O’Gorman (2020) suggested that vertical stability changes may contribute significantly to subtropical extreme ascent decreases, and Mpanza and Tandon (2022) suggested that changes in differential vorticity advection play a key role.

These proposed mechanisms may be related in that vertical stability influences the eddy length scale (as captured by the standard formulation for Rossby deformation radius), and eddy length scale would influence the curvature of geopotential surfaces, and hence the vorticity. Thus, the horizontal scale of EPEs are potentially key to the theoretical understanding of extreme ascent changes. Furthermore, EPE horizontal scale is a fundamental feature of a precipitating weather system that, in the extratropics, directly relates to the advective time scale of the system and thus the accumulated precipitation (e.g., Dwyer & O’Gorman, 2017).

Thus, beyond their connections with extreme ascent, EPE horizontal scales and their behavior in climate models can provide an important characterization of EPEs. In this study, we perform such a characterization in modern GCMs, focusing on the annual maximum of daily precipitation. Our analysis reveals significant spread in EPE horizontal scale among models. This finding raises the central question motivating this study: what is the influence of horizontal model resolution on the horizontal scale of EPEs?

To address this question, we compute the horizontal scales of EPEs (and other relevant quantities) for 27 GCMs participating in the Coupled Model Intercomparison Project Phase 6 (CMIP6), and the results show a clear model resolution influence on the horizontal scales of EPEs as well as extreme ascent. A few earlier studies have also documented the resolution dependence of extreme precipitation over particular regions (Liang et al., 2023; Scher et al., 2017; van der Wiel et al., 2016). However, the current study reveals a more general resolution dependence in simulated EPEs that is not specific to particular regions or lower boundary features. Such resolution dependence is especially concerning because of the possible impact on not just the spatial details, but also the intensity of EPEs.

In Section 2, we detail our data sources and methodology. In Section 3, we present our results regarding the horizontal scales computed using several different quantities, along with their resolution dependence. We also assess the roles of planetary‐scale dynamics and parameterized processes in determining the horizontal scales. We close with some concluding remarks in Section 4.

## 2 Methods

### 2.1 EPE Definition and Data Sets

The GCM output analyzed in this study comes from two sub‐projects of CMIP6: (a) CMIP, which includes GCMs running at standard resolution (Eyring et al., 2016), and (b) the High Resolution Model Intercomparison Project (HighResMIP, Haarsma et al., 2016), which includes high resolution models along with coarser resolution variants of those models. Unless otherwise stated, we will hereafter use “CMIP” to refer to the CMIP sub‐project of CMIP6. In this study, we analyze the following four quantities (with the CMIP6 variable name given in parentheses): total precipitation (pr), vertical velocity (wap), parameterized “convective” precipitation (prc), and geopotential height (zg). We compute geopotential from geopotential height, and we compute resolved (“large scale”) precipitation as the difference of pr and prc.

Vertical velocity and geopotential are analyzed at the 500hPa pressure level. This level is chosen because pressure levels closer to the surface include too many topographical effects, while pressure levels at higher altitudes are sometimes too close to or above the tropopause. Unless otherwise stated, all computations are performed using model output at daily resolution because many models do not report data at higher temporal resolution, and it is common to investigate EPEs using daily data (e.g., O’Gorman & Schneider, 2009; Pfahl et al., 2017; Tandon et al., 2018).

Table 1 lists the models analyzed in this study and the variables available from each model. We limited our analysis to models for which daily output of both wap and pr were available during the historical period and for which wap and pr were reported on the same grid. Six models provided daily wap files, but an excessive amount of data within the files was missing, and so these models were not included in our analysis. All of the models we analyze use a standard spherical polar grid in the atmospheric component.

**Table 1:** The Models and Variables Analyzed in This Study

Model name	CMIP6 sub‐project	# latitude points	# longitude points	Nominal resolution (km)	Available variables			
					pr	wap	prc	zg
ACCESS‐CM2	CMIP	144	192	250	X	X	X	X
BCC‐CSM2‐HR	HighResMIP	400	800	50	X	X	X	X
CanESM5	CMIP	64	128	500	X	X	X	X
CESM2	CMIP	192	288	100	X	X	X	X
CESM2‐FV2	CMIP	96	144	250	X	X	X	X
CESM2‐WACCM	CMIP	192	288	100	X	X	X	X
CESM2‐WACCM‐FV2	CMIP	96	144	250	X	X	X	X
CMCC‐CM2‐HR4	HighResMIP	192	288	100	X	X	X	X
CMCC‐CM2‐VHR4	HighResMIP	768	1152	25	X	X		X
EC‐Earth3	CMIP	256	512	100	X	X	X	X
ECMWF‐IFS‐HR	HighResMIP	361	720	25	X	X	X	X
ECMWF‐IFS‐LR	HighResMIP	181	360	50	X	X	X	X
ECMWF‐IFS‐MR	HighResMIP	181	360	50	X	X	X	X
GFDL‐CM4	CMIP	90	144	250	X	X		X
HadGEM3‐GC31‐HH	HighResMIP	768	1024	50	X	X	X	
HadGEM3‐GC31‐HM	HighResMIP	768	1024	50	X	X	X	
HadGEM3‐GC31‐LL	HighResMIP	144	192	250	X	X	X	
HadGEM3‐GC31‐MM	HighResMIP	324	432	100	X	X	X	
HiRAM‐SIT‐HR	HighResMIP	768	1536	25	X	X	X	X
IITM‐ESM	CMIP	94	192	250	X	X	X	X
INM‐CM4‐8	CMIP	120	180	100	X	X	X	X
INM‐CM5‐0	CMIP	120	180	100	X	X	X	X
MPI‐ESM‐1‐2‐HAM	CMIP	96	192	250	X	X	X	X
MPI‐ESM1‐2‐HR	HighResMIP	192	384	100	X	X	X	X
MPI‐ESM1‐2‐LR	CMIP	96	192	250	X	X	X	X
MPI‐ESM1‐2‐XR	HighResMIP	384	768	50	X	X	X	X
TaiESM1	CMIP	192	288	100	X	X	X	X

*Note.* Quantities are referred to by their CMIP6 variable name (wap, pr, prc and zg are vertical velocity, total precipitation, parameterized precipitation and geopotential, respectively). We use output from the “historical” scenario for all CMIP models and the “hist‐1950” scenario for all HighResMIP models. The nominal resolution given is for the atmospheric component of the model, and the nominal resolution is the same as the native nominal resolution for all models in this study.

Under CMIP6, each model stores the attributes “nominal resolution” (the approximate horizontal resolution of the grid on which data are stored) and “native nominal resolution” (the approximate native horizontal resolution of the model). Table 1 shows the nominal resolution for the atmospheric component of each model, and for each model we analyze in this study, the native nominal resolution matches the nominal resolution, and hereafter, we simply use the term “nominal resolution.” The models span a range of nominal resolutions, from 25 km for three models (CMCC‐CM2‐VHR4, ECMWF‐IFS‐HR and HiRAM‐SIT‐HR) to 500 km for CanESM5. It should be noted, however, that the nominal resolution is not precise, since it is based on the global average of the diagonal distance across each grid cell, and it is binned by ranges of resolution to facilitate grouping of models with similar resolutions (Taylor et al., 2018, Appendix 2). Thus, Table 1 also shows the exact number of latitude and longitude points in each model, which gives a more precise indication of the horizontal model resolution, and we will use a more precise measure of horizontal resolution when assessing resolution dependence below.

For a number of models, multiple ensemble members were available for which each ensemble member differed only by its initial conditions. Averaging across ensemble members can reduce noise due to internal climate variability, but to maintain consistent sampling across models, we use only one ensemble member for each model. Using one model (MPI‐ESM‐1‐2‐HAM), we have tested for sensitivity to the choice of ensemble member, and our results did not significantly change when a different ensemble member was used (not shown).

In this study, a 1‐year return period is used with an EPE defined as the event in the return period with the maximum daily accumulated precipitation for each location. A 1‐year return period was used to allow for sufficient sampling of the events in the 20‐year study period (1981–2000) and to facilitate comparison with earlier studies that commonly use this approach and study period (e.g., Pfahl et al., 2017; Tandon et al., 2018). Thus, in each model, each location provides one EPE per year, for 20 total EPEs during the study period. In these computations, we have included “dry days,” for which precipitation is so low that the day can be considered dry. We have tested setting the precipitation on dry days (precipitation less than 1 mm $d^{-1}$) to be missing, effectively excluding horizontal scale calculations for any EPE that is less than 1 mm $d^{-1}$. Our results were not significantly different when we treated dry days in this way.

We also analyze precipitation from three satellite‐based observational products: CMORPH (Xie et al., 2019), IMERG (Huffman et al., 2019), and GPCP version 3.2 (Huffman et al., 2022). CMORPH uses passive microwave measurements on low‐earth orbiting satellites and infrared brightness temperature data from geostationary satellites. CMORPH data are provided on an 8km×8km grid over 60°S to 60°N, with temporal resolution from monthly up to 30 min from 1 January 1998 to the present. IMERG uses NASA and JAXA's Global Precipitation Measurement (GPM) satellite constellation to produce a precipitation data set. IMERG data are provided on a 0.1°×0.1° grid over the entire globe, with temporal resolution of monthly up to 30 min from June 2000 to the present. We used daily precipitation for both CMORPH and IMERG. GPCP uses measurements from the same satellites as IMERG (but with different processing algorithms) along with land‐based rain gauge measurements to produce a data set on a 0.5°×0.5° grid over the entire globe with daily temporal resolution from June 2000 to the present.

While these three observational products are distinct, they are somewhat interdependent as GPCP v3.2 uses IMERG as input over the 55°N to 55°S latitude range (Huffman et al., 2022), and CMORPH uses GPCP for bias correction over the ocean (Xie et al., 2019). These observational products are distinct from reanalysis systems that assimilate observations into general circulation models in order to estimate observations. We did not examine reanalysis products in this study as the general circulation models that they rely on are potentially subject to the same resolution dependence as the CMIP6 models. We wished to rule out this possible confounding factor when comparing models and observations. For all three observational products examined in this study, we analyzed data over 2001–2014. Though the models were analyzed over 1981–2000 (to facilitate comparison with earlier studies), we have performed sensitivity tests (shown further below) demonstrating that extreme precipitation horizontal scales computed over 1981–2000 differed little from those computed over 2001–2014. Thus, model results from 1981–2000 can be reasonably compared with observational results from 2001–2014.

### 2.2 Horizontal Scale Computation

Horizontal scales were computed following the method of Mpanza and Tandon (2022) as follows:

1. For each grid cell in each model or observational product, the day of annual maximum precipitation is determined for each year during the study period. (That is, EPE dates are determined internally in each model or observational product, and the EPE dates in models are not expected to be the same as in the observational products.) To compute anomalies, we subtract the climatology from the full field. This climatology is computed as the average over the study period of the 3‐month seasonal average centered around the month during which the EPE occurs.
2. The horizontal scale of an anomaly is computed by first determining the e‐folding distances of the anomaly in each cardinal direction. The e‐folding distance is the distance from the grid cell of interest at which the anomaly decreases by a factor of e. Linear interpolation is applied between grid cell centers when performing this computation.
3. The east and west e‐folding distances are averaged to determine a zonal e‐folding distance, and the north and south e‐folding distances are averaged to determine a meridional e‐folding distance.
4. The zonal and meridional e‐folding distances are divided by 0.19×2π=1.19 to determine their scales in standard inverse wavenumber form. This scaling follows Barnes and Hartmann (2012), who showed that the e‐folding distance of a cosine wave is 0.19 times its wavelength. For events whose computed zonal or meridional length scale is below the grid spacing of the model or observational product, the length scale is set to the grid spacing. This adjustment has no significant effect on our results, as almost all EPEs in all models and observational products have zonal and meridional scales greater than the model resolution.
5. The zonal and meridional wavenumbers are combined using a Euclidean norm. Specifically, we compute

(1) $$L={\left(L_{x}^{-2}+L_{y}^{-2}\right)}^{-\frac{1}{2}},$$

where L is the horizontal scale with zonal component $L_{x}$ and meridional component $L_{y}$.

For a given quantity in a given model or observational product, this computation is repeated for each year and the results are averaged over the study period to produce a composite horizontal scale at a given grid point. We perform this computation on the native grid of each model. As will be discussed in more detail below, our results are not strongly affected if we interpolate the model output to a standard grid prior to computing the horizontal scale.

**Figure 1:**
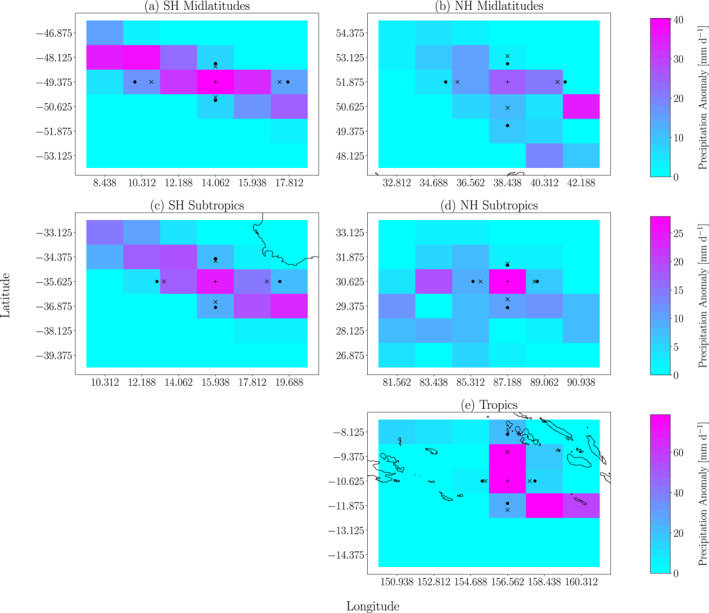
Examples illustrating the computations of zonal and meridional length scales for extreme precipitation events (EPEs) in a historical simulation of ACCESS‐CM2 on (a) 16 April 1992 in the SH midlatitudes, (b) 17 April 1985 in the NH midlatitudes, (c) 7 October 1987 in the SH subtropics, (d) 23 July 1991 in the NH subtropics and (e) 16 February 1986 in the tropics. The precipitation anomaly field is shown in shading, and the shading scales used in each row are different. The center of the cell where the EPE is occurring is indicated with a plus sign. Circles indicate the e‐folding distances in the four cardinal directions. Crosses indicate the locations that are distances $L_{x}$ (in the zonal direction) and $L_{y}$ (in the meridional direction) from the EPE location. Note that the length scale along a particular coordinate is computed after averaging the two e‐folding length scales along that coordinate. See the text for additional details.

Figure 1 shows examples of $L_{x}$ and $L_{y}$ calculations for EPEs in different locations. Notice that the precipitation anomalies are slanted northwest‐to‐southeast in the Southern Hemisphere (SH). This is a common structure for EPEs in the extratropics, where extreme precipitation often occurs with warm air advection on the leading edge of a cyclone. In the Northern Hemisphere (NH), EPEs with southwest‐to‐northeast slant are common for the same reason, although such structures are not as apparent in the specific examples shown in Figure 1. One might consider using alternative definitions of length scale that better describe the spatial structure along these slanted axes. However, we have opted in this study to use the horizontal scales along the cardinal directions since these would directly enter into theoretical formulations in the standard meteorological reference frame and thus facilitate reproducibility and comparison with theoretical expectations.

## 3 Results

### 3.1 Horizontal Scales of EPEs

**Figure 2:**
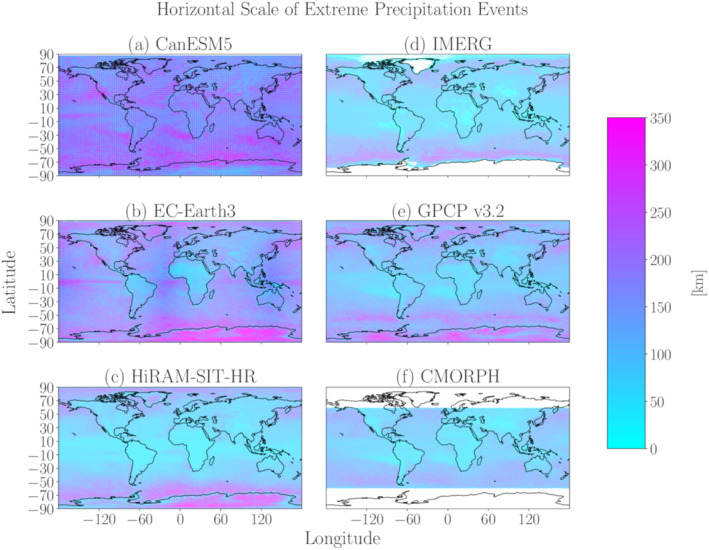
Global plots of the precipitation anomaly horizontal scales averaged over EPEs during 1981–2000 for the models (a) CanESM5, (b) EC‐Earth3 and (c) HiRAM‐SIT‐HR and averaged over 2001–2014 for the observational products (d) IMERG, (e) GPCP v3.2 and (f) CMORPH. Locations with missing data are shaded white.

To get an initial sense of the range of horizontal scales produced by models, Figure 2 shows the horizontal scale of EPEs for CanESM5, EC‐Earth3 and HiRAM‐SIT‐HR, representing examples of low, mid and high‐resolution models, respectively. These models produce qualitatively similar results over the extratropics, with peak horizontal scales over the midlatitude and high latitude oceans. The coarser‐resolution models (CanESM5 and EC‐Earth3) also produce a local peak over the equatorial oceans that is not apparent in HiRAM‐SIT‐HR. For all three models, the horizontal scales are generally lower over land than over ocean.

The EPE horizontal scales in Figure 2 correspond well with those obtained by Dwyer and O’Gorman (2017), despite the differences in methodology. In particular, Dwyer and O’Gorman (2017) exclusively use the zonal length scale of the precipitation field, while we consider both the zonal and meridional horizontal scales. Dwyer and O’Gorman (2017) also determined horizontal scales based on exceedance of 25% of the 99.9th percentile of precipitation, instead of the e‐folding distance used in the present study. Moreover, Dwyer and O’Gorman (2017) focused on winter averages and used model output at 3‐hourly and hourly temporal resolution, rather than analyzing the full year at daily resolution. Altogether, this comparison suggests that our computations of horizontal scale are not strongly sensitive to the methodology details.

Despite some qualitative similarity in the horizontal scale patterns, there are notable quantitative differences between the models. For example, the horizontal scales over the SH midlatitudes are around 250 km in CanESM5 but around 150 km in EC‐Earth3 and 100 km HiRAM‐SIT‐HR. In the tropics, the horizontal scales are below 100 km in HiRAM‐SIT‐HR, but are 200–300 km in CanESM5 and EC‐Earth3. This spread in horizontal scales motivates further investigation into the differences between the models.

Figures 2d–2f show EPE horizontal scales from the three observational products selected for this study. These data sets qualitatively agree with each other, although the horizontal scales from GPCP are generally higher than those from CMORPH and IMERG. Of the models included in Figure 2, the highest resolution model (HiRAM‐SIT‐HR) shows the best agreement with the observational products, whereas the other coarser‐resolution models produce horizontal scales that are generally higher than those from observations. As explained in the Methods, we have chosen a different averaging period for the observational products (2001–2014) than for the models (1981–2000), and we will show below that our results are not sensitive to this choice of averaging period.

**Figure 3:**
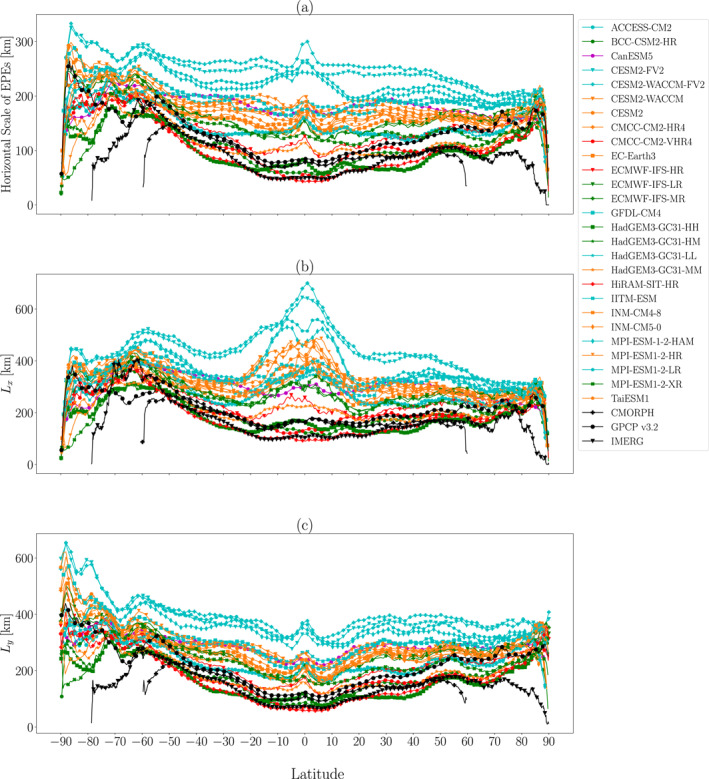
Zonal mean (a) horizontal scale, (b) $L_{x}$, and (c) $L_{y}$ for EPEs during 1981–2000 for models and during 2001–2014 for observational products. The vertical scales of the panels are different. In this and subsequent figures, markers and lines are colored in order to group models into the following ranges of nominal resolution: (brown) 500 km, (green) 250 km, (orange) 100 km, (blue) 50 km and (red) 25 km. See the Section 2 for additional information about the nominal resolutions of the models.

How do the models' EPE horizontal scales vary in the zonal average? Figure 3a shows local maxima in horizontal scales near the poles and in the southern midlatitudes, as was apparent in Figure 2. In the coarser‐resolution models, there is also a local peak at the equator that is less apparent in the higher resolution models and observational products. Furthermore, for all models, the horizontal scales sharply plummet near the poles. It is somewhat surprising that the higher resolution models and observational products produce higher horizontal scales in the midlatitudes than in the tropics, as common theories predict higher eddy length scale in the tropics than in the extratropics (e.g., Frierson et al., 2006). A possible reason for this contrast is that tropical EPEs are governed more by convection with smaller horizontal scales compared to the larger‐scale dynamical variations that influence extratropical EPEs.

Over the tropics and subtropics, the three observational products generally agree with each other, and they generally agree with values produced by the higher resolution models. Howeever, the observational products diverge in the midlatitudes, and much of this divergence is due to sharp drops in horizontal scales near the southern edges of each data set's domain. This could be due to grid point–scale spatial noise toward the edges of the observational domains, a matter requiring further investigation.

To gain additional insight, Figures 3b and 3c show the $L_{x}$ and $L_{y}$ components of the horizontal scale, respectively. About a third of the models analyzed produce global maximum $L_{x}$ values at the equator, whereas the remaining models (as well as the three observational products) produce maximum $L_{x}$ values in the southern extratropics. Almost all of the models produce local maximum $L_{y}$ values at the equator, although as with $L_{x}$, this local maximum is less apparent in higher resolution models and the observational products. Near the poles, $L_{x}$ in each model plummets, behavior also apparent in L, whereas this behavior is less apparent in $L_{y}$ (except in IMERG). This result raises the possibility that the zonal resolution, which increases near the poles as the zonal grid spacing decreases, may be influencing the horizontal scale, and we will explore this possibility further below. In the midlatitudes, $L_{x}$ and $L_{y}$ are similar for each model and observational product, and this is also true for observations and higher resolution models in the tropics and subtropics. However, for reasons that are unclear, coarser‐resolution models produce $L_{x}$ and $L_{y}$ that are very different at lower latitudes.

Overall, there is a clear spread among the models' EPE horizontal scales, which again motivates further investigation into the differences between the models. The models appear to show the most spread near the equator and less spread over subpolar latitudes. For example, near 70°S the models have a spread of around 120km (ranging from around 170km to 290km), and near 70°N the models have a similar spread of approximately 130km (ranging from around 90km to 220km). In contrast, near the equator the models have a much larger spread of about 260km (ranging from around 40km to 300km). Thus, the horizontal scale varies among models by approximately a factor of two over subpolar latitudes and approximately a factor of 7 at the equator. In contrast with the models, the spread among observational products is lower in the tropics than it is in the midlatitudes. This contrast is expected, as the observations are more constrained by precipitation measurements such as TRMM in the tropics than they are in the extratropics.

**Figure 4:**
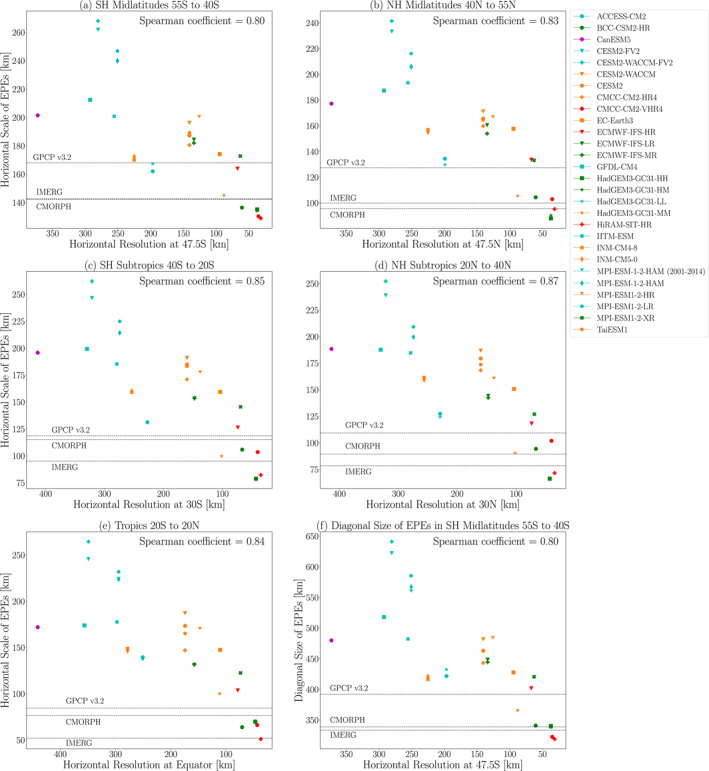
Horizontal scales of EPEs versus model resolution during 1981–2000 averaged over the (a) SH midlatitudes, 55°S–40°S, (b) NH midlatitudes, 40°N–55°N, (c) SH subtropics, 40°S–20°S, (d) NH subtropics, 20°N–40°N and (e) tropics 20°S–20°N. The horizontal axis shows the model resolution at the midpoint of the latitude band of interest arranged in order of decreasing grid box size, that is, increasing model resolution. (f) As in (a), except the vertical axis shows the diagonal size of the EPEs, computed as described in the text. The horizontal lines show the EPE horizontal scales from three observational products (CMORPH, IMERG and GPCP v3.2) averaged over EPEs during 2001–2014. Note that in panel a, the lines for CMORPH and IMERG are indistinguishable. As a sensitivity test, the 2001–2014 EPE horizontal scale for MPI‐ESM‐1‐2‐HAM is also shown. The intermodel Spearman correlation coefficient is indicated in the upper right of each panel. The vertical scales of the panels are different.

To what extent is horizontal model resolution influencing this sizable intermodel spread in horizontal scale? To address this question, Figure 4a compares the extreme ascent horizontal scales for each model in the 55–40°S latitude range with its horizontal resolution. We chose this latitude range for much of our analysis because it is mostly ocean‐covered, allowing us to isolate resolution effects that are independent of specific surface features, like mountains and western boundary currents. Here and in subsequent figures, the horizontal resolution is computed as the diagonal distance across a grid cell at the midpoint of the latitude range being examined (47.5°S in this case).

Figure 4a shows a clear relationship between the EPE horizontal scales and the model resolution: the horizontal scale decreases as model resolution increases. For approximately a factor of seven decrease in the distance across a grid cell, the horizontal scale decreases by approximately a factor of two, with a Spearman correlation coefficient of 0.80. There is some scatter in the horizontal scale–resolution relationship, indicating that other factors also influence horizontal scale. Those other factors might be differences in vertical stability during EPEs, parameterization schemes and physics packages. Thus, although Figure 4a shows a clear influence of model resolution on EPE horizontal scales, model resolution is not the only influence, and other influences should be examined in future studies.

The computed horizontal scales in Figure 4a are comparable, and for some models lower, than the horizontal resolution, despite the fact that we have not allowed for $L_{x}$ and $L_{y}$ values that are lower than the model grid spacing (see Section 2). Such seemingly puzzling results are possible because the way the zonal and meridional components are combined when computing the horizontal resolution is different from how they are combined when computing L. In particular, the horizontal resolution, $L_{g}$, is computed as the diagonal distance across the grid cell, that is,

(2) $$L_{g}=\sqrt{L_{gx}^{2}+L_{gy}^{2}},$$

where $L_{gx}$ and $L_{gy}$ are the zonal and meridional grid spacings, respectively. This is the standard method used in CMIP6, but this approach contrasts with how zonal and meridional inverse wavenumbers are typically combined, as in Equation 1, which is the approach we used for computing horizontal scale.

For a more apples‐to‐apples comparison, we should instead compute the diagonal size, $L_{d}$, of the vertical velocity anomalies in a manner analogous to Equation 2, that is, $L_{d}=\sqrt{L_{x}^{2}+L_{y}^{2}}$. Figure 4f plots the diagonal size of EPEs versus the horizontal resolution, and this shows the same resolution dependence as in Figure 4a, but the diagonal size is about 2.5 times larger than the horizontal scale computed using Equation 1, and this diagonal size is clearly larger than the model resolution for all models. Hereafter, we will continue to show horizontal scales computed using Equation 1, since this approach facilitates comparison with earlier studies and theoretical expectations. However, readers should keep in mind that the diagonal sizes of EPEs are approximately 2.5 times larger than the inverse wavenumber–based horizontal scale.

The resolution‐dependence of EPE horizontal scale is also apparent in Figure 4 when comparing results for models that are nearly identical except for horizontal resolution. For example, CESM2 (Zhu et al., 2022) has a resolution of approximately 140km and an EPE horizontal scale around 190km, while CESM2‐FV2 has a resolution of approximately 280km and a horizontal scale around 265km. Similarly, MPI‐ESM1‐2‐HR (Gutjahr et al., 2019) has a resolution of approximately 120km and a horizontal scale around 200km, while MPI‐ESM1‐2‐LR has a resolution of 250km and a horizontal scale around 250km. Altogether, these model variants produce decreasing horizontal scale with increasing model resolution, reiterating the overall pattern revealed by our multimodel analysis.

How do the EPE horizontal scales in models compare with observational estimates? Figure 4a includes the EPE horizontal scales computed from three observational products, CMORPH, IMERG, and GPCP v3.2 over the 2001–2014 period. (See Section 2 for additional details.) The horizontal scales for CMORPH and IMERG are very similar (around 143km), whereas GPCP v3.2's horizontal scale is approximately 168km. As mentioned in the Methods, these observational estimates are interdependent, and so they are expected to produce similar (though not identical) results.

The EPE horizontal scales from ACCESS‐CM2, HadGEM3‐GC31‐LL, HadGEM3‐GC31‐MM and ECMWF‐IFS‐HR (with nominal resolutions of 250km, 250km, 100km, and 25km, respectively) all fall within the range produced by the observational products. If the difference between the observational estimates (approximately 25 km) is adopted as a measure of the observational uncertainty, then one could argue that any horizontal scale that is within 25 km of any of the observational estimates is within the range of observational uncertainty. By this measure, all but two of the models with grid spacing greater than 240 km fall within the range of observational uncertainty. Thus, in general, high model resolution is not a requirement in order to produce realistic EPE horizontal scales in the SH midlatitudes, although higher resolution is surely beneficial when attempting to simulate EPEs that are influenced by localized surface features (e.g., Scher et al., 2017).

As mentioned in the Methods, the study period used for the observational estimates (2001–2014) is different from that used for the models (1981–2000). To assess possible sensitivity to the choice of time period, Figure 4 also includes a second point for the MPI‐ESM‐1‐2‐HAM model, corresponding to model results averaged over 2001–2014. For both time periods, MPI‐ESM‐1‐2‐HAM produces nearly the same horizontal scale (approximately 240 km), and the difference due to time period is much less than the intermodel differences due to resolution. These results indicate that the precise choice of time period has little influence on the horizontal scale, and it is reasonable to compare model results computed over 1981–2000 to observational estimates computed over 2001–2014.

Figures 4b–4e show that the resolution dependence apparent in the SH midlatitudes is also clearly apparent, and with higher intermodel correlations, over other latitude bands. The strongest intermodel correlations are in the subtropics (0.85 in the SH subtropics and 0.87 in the NH subtropics). As over the SH midlatitudes, the higher resolution models (though not necessarily the highest resolution models) generally produce better agreement with observational estimates. In the tropics, the benefit of higher resolution is especially clear, as the mid‐resolution models do not fall within the range of observational uncertainty. Furthermore, the sensitivity to resolution is stronger in the tropics and subtropics compared to the midlatitudes: for a factor of seven increase in model resolution, the EPE horizontal scale decreases by approximately a factor of three in the subtropics and a factor of five in the tropics. Despite these different sensitivities, the intermodel correlations in all of the analyzed latitude bands are quite similar, falling within the range 0.8–0.9. Thus, in much of our more detailed analysis below, we focus on the SH midlatitudes with the expectation that our findings are applicable in other latitude bands. However, more detailed analysis of other latitude bands should be conducted in the future in order to confirm the reasons for resolution dependence across all regions.

### 3.2 Horizontal Scales of Vertical Velocity Anomalies

**Figure 5:**
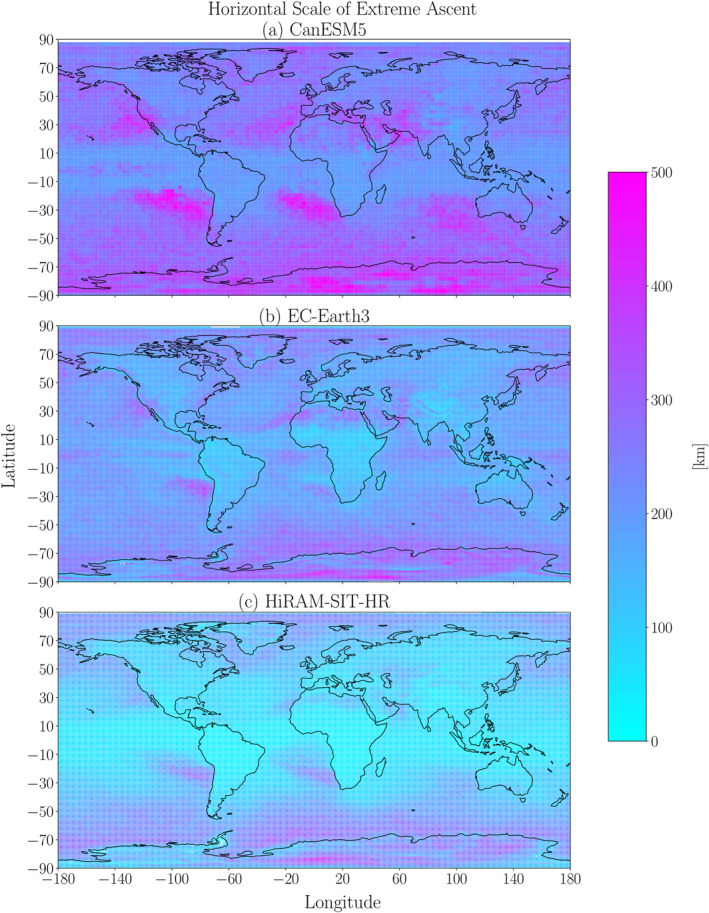
Global plots of the 500hPa vertical velocity anomaly horizontal scales for annual maximum EPEs averaged over 1981–2000 for (a) CanESM5, (b) EC‐Earth3 and (c) HiRAM‐SIT‐HR.

As vertical velocity is a key driver of extreme precipitation (e.g., O’Gorman & Schneider, 2009), the horizontal scales of vertical velocity anomalies during EPEs (i.e., the extreme ascent horizontal scales) are expected to follow similar behavior as EPE spatial scales. To demonstrate this correspondence, Figure 5 shows global plots of extreme ascent horizontal scales for CanESM5, EC‐Earth3 and HiRAM‐SIT‐HR, respectively, which capture the range of model behavior. Qualitatively, the distribution of extreme ascent horizontal scales is similar to that of the EPEs themselves, with peak length scales over the midlatitude storm tracks and (in coarser‐resolution models) the equatorial oceans. In contrast with the EPE horizontal scales, the extreme ascent horizontal scales also show local peaks over the subtropical dry zones, off the west coasts of the continents.

The Spearman spatial correlations between the extreme ascent and EPE spatial scales range from 0.56 for CanESM5 to 0.91 for HiRAM‐SIT‐HR, without any clear dependence of this spatial correlation on resolution. These generally high correlations show that extreme ascent influences extreme precipitation not just in terms of intensity (e.g., Pfahl et al., 2017) but also in terms of horizontal scale. Quantitatively, however, the EPE horizontal scales are approximately 25% smaller than the extreme ascent horizontal scales. This difference may be due to the fact that ascending motion does not always produce precipitation, and thus the region over which ascending motion occurs would be expected to be larger than the region over which precipitation occurs.

Moreover, the models do not show a straightforward correspondence between extreme ascent strength (not shown) and extreme ascent horizontal scale. For example, horizontal scales are generally large in both the subtropical dry zones and the extratropics, but EPI and extreme ascent strength are relatively low in the subtropical dry zones and stronger in the extratropics (Tandon et al., 2018). Such complexity may be due to competing effects of horizontal scale. On the one hand, a larger spatial scale can weaken the coupling between convection and the large scale circulation, thus reducing extreme ascent strength (Nie & Sobel, 2016; Tandon et al., 2018). On the other hand, an extratropical cyclonic eddy would typically be associated with warm air advection on its eastern flank, which is favorable to ascending motion (e.g., Binder et al., 2017). So it is possible that larger spatial scales in the extratropics are more favorable to stronger warm air advection and extreme ascent, and additional work is needed to investigate this possibility.

**Figure 6:**
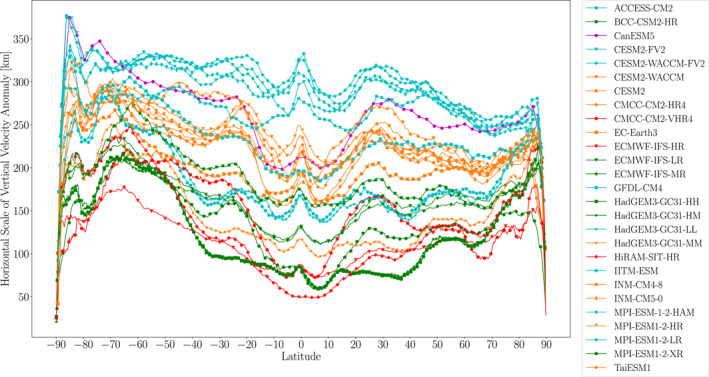
Zonal mean horizontal scales of vertical velocity anomalies at 500 hPa averaged over EPEs during 1981–2000.

As with the models' EPE horizontal scales, the extreme ascent scales' zonal averages can be considered. For almost all models, the extreme ascent horizontal scales in Figure 6 show local maxima over the equator, the subtropics, subpolar regions, southern midlatitudes. This meridional dependence resembles that of the EPE horizontal scales (Figure 3). However, in contrast with the EPE horizontal scale, the extreme ascent horizontal scale in most models shows a clearer local maximum in the subtropics in the zonal average, reiterating the overall stronger local maximum in extreme ascent horizontal scale which was apparent in the global plots of Figure 5. As with the EPE horizontal scales, there is a clear spread among the models' extreme ascent horizontal scales, with greater spread for values near the equator and less spread at higher latitudes.

Frierson et al. (2006) showed qualitatively similar meridional structure when examining eddy length scale computed by Fourier analysis of meridional velocity (without limiting to EPEs) in simulations with an idealized moist general circulation model, especially the localized peaks in length scale at the equator and over the subtropical and subpolar regions, with a sharp drop in length scale toward the poles. Kidston et al. (2010) analyzed eddy length scale in CMIP3 models using Fourier analysis of meridional velocity (again without limiting to EPEs), and some models showed localized peaks at the equator and in the subtropics similar to our results, but several of the models analyzed did not show local maxima in the subtropics.

**Figure 7:**
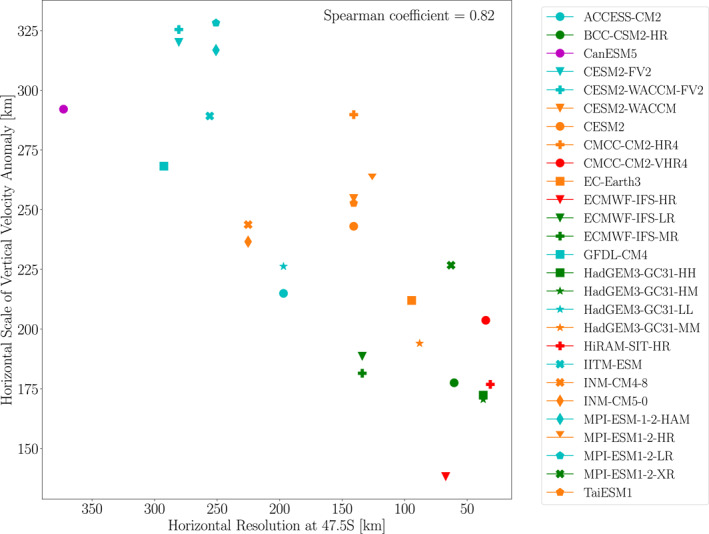
Scatter plot of the horizontal scale of vertical velocity anomalies at 500 hPa averaged over all EPEs during 1981–2000 and averaged over the 55°S to 40°S latitude range plotted versus the model horizontal resolution at 47.5°S. The Spearman correlation coefficient is indicated in the upper right of the figure.

Quantitatively, however, the extreme ascent horizontal scales we obtain are approximately one third of those implied by the results of Frierson et al. (2006) and Kidston et al. (2010). We will revisit this contrast below after discussing horizontal scales of geopotential anomalies. [Note that Frierson et al. (2006) and Kidston et al. (2010) calculated horizontal scale in terms of wavelength, which needs to be divided by 2π when quantitatively comparing with the inverse wavenumbers that we compute.] In contrast with the hemispherically symmetric results in the aquaplanet simulations of Frierson et al. (2006), and in qualitative agreement with the results of Kidston et al. (2010), the horizontal scales in our Figure 6 are generally higher in the southern extratropics than in the northern extratropics. Furthermore, Figure 7 shows a similar resolution dependence as for EPE horizontal scale: the extreme ascent horizontal scale decreases as model resolution increases, with an intermodel correlation of 0.82.

### 3.3 Roles of Resolved Versus Parameterized Precipitation

The above analysis motivates further investigation of the reasons for the resolution dependence. One question that arises is whether the simulated EPE spatial scales are being determined by resolved or parameterized convection. But before addressing this point, some clarification regarding terminology is called for. In climate models that parameterize convection, total precipitation in a given grid cell is the sum of the precipitation that is resolved on the model grid and the parameterized precipitation meant to capture the effects of subgrid‐scale processes. In the modeling community, both forms of precipitation are referred to with various terms, which can create confusion. For example, in CMIP6 archives and documentation, parameterized precipitation is referred to as “convective precipitation,” even though it does not include precipitation due to grid‐scale convection, which is potentially an important consideration for extreme precipitation. Moreover, parameterized precipitation is sometimes also referred to as “cumulus precipitation” even though not all parameterized precipitation is due to cumulus convection. On the other hand, precipitation resolved at the grid scale is often referred to as “large‐scale” or “stratiform” precipitation even though “large‐scale” is vague and potentially misleading (since a typical 100 km grid cell is not large‐scale compared to large‐scale atmospheric circulation features) and not all resolved precipitation is stratiform. Furthermore, vertical momentum transport by the convective parameterization (which is especially important in the tropics) can contribute to resolved precipitation, and so the physical processes driving parameterized and resolved precipitation are not as independent as some of the aforementioned terms might imply. Thus, to avoid confusion, the two forms of precipitation are referred to hereafter simply as “resolved precipitation” and “parameterized precipitation.”

**Figure 8:**
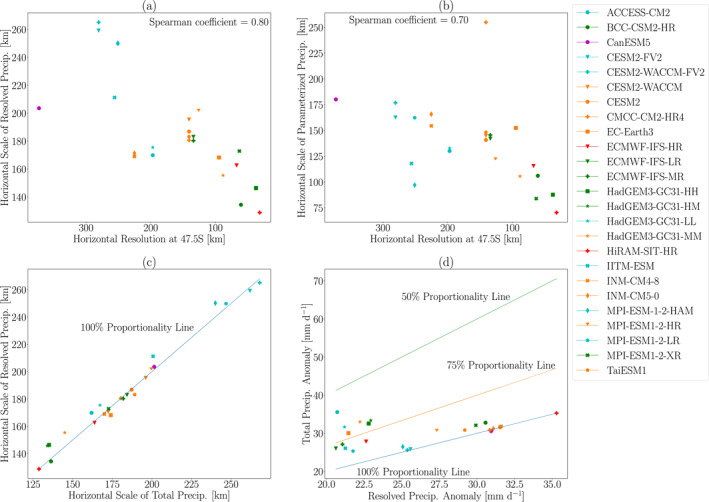
The horizontal scale of (a) resolved precipitation anomalies and (b) parameterized precipitation anomalies plotted versus horizontal resolution at 47.5°S. The Spearman correlation coefficient is indicated in the upper right of panels (a, b). (c) The horizontal scale of resolved precipitation anomalies versus the horizontal scale of total precipitation anomalies. For reference, a solid line indicating a one‐to‐one relationship is also shown. (d) The total precipitation anomaly (i.e., the extreme precipitation intensity, EPI) versus the resolved precipitation anomaly. For reference, solid lines indicating EPI that is 50%, 75% and 100% resolved precipitation are shown. All quantities in this figure are averaged over EPEs in the 55°S to 40°S latitude range during 1981–2000.

So to what extent is the EPE horizontal scale determined by resolved versus parameterized precipitation? Figure 8a shows the horizontal scales of resolved precipitation, which reveals that the resolved precipitation horizontal scales exhibit a similar resolution dependence to the horizontal scales for total precipitation and vertical velocity: the horizontal scale decreases as model resolution increases. Furthermore, the resolved precipitation horizontal scale for each model is quantitatively very similar to the total precipitation horizontal scale (cf. Figure 4). Figure 8b shows the parameterized precipitation horizontal scale versus model resolution. Here there is also an indication of a model resolution effect, but the intermodel correlation (0.70) is not as strong as for the resolved precipitation horizontal scales. Furthermore, the parameterized precipitation horizontal scales are 30%–50% lower than the total precipitation horizontal scales. The precise reasons for this resolution‐dependence of parameterized precipitation require further investigation, but if parameterized precipitation is contributing even a small amount to the total precipitation, then as the spatial scale of the overall EPE changes with resolution, we would expect the spatial scale of the parameterized precipitation to also change with resolution. This matter aside, our analysis suggests that EPE horizontal scales are overwhelmingly determined by resolved rather than parameterized precipitation.

Figure 8c shows the total precipitation horizontal scale versus the resolved precipitation horizontal scale, which reiterates the close correspondence between the two quantities, as the horizontal scales for all models fall very close to the one‐to‐one line. Some models in Figure 8c appear to have slightly greater resolved precipitation horizontal scales than total precipitation horizontal scales. For example, IITM‐ESM has a total precipitation horizontal scale around 201km and a resolved precipitation horizontal scale around 211km, and MPI‐ESM‐1‐2‐HAM has a total precipitation horizontal scale around 240km but a resolved precipitation horizontal scale around 250km. These differences are not surprising because precipitation horizontal scale and precipitation intensity do not have a straightforward relationship, and the spatial scale of resolved precipitation can be larger than the spatial scale of the total precipitation even though the intensity of resolved precipitation is constrained to be no greater than the intensity of total precipitation.

Figure 8d shows that resolved precipitation accounts for more than 50% of the total EPI in all of the models, and for nearly half of the models, this proportion is close to 100%. For models with the highest EPI (precipitation anomaly over $24\;\text{mm}\;d^{-1}$), almost all of their precipitation is due to resolved precipitation. There is no clear influence of model resolution on the proportion of resolved precipitation. For example, HiRAM‐SIT‐HR (a high resolution model), CanESM5 (a coarse resolution model), and several mid‐resolution models produce EPEs with approximately 100% resolved precipitation. Furthermore, both coarser and higher resolution models fall in the 50%–75% range. Thus, the dominance of resolved precipitation over parameterized precipitation likely has more to do with the model physics and parameterizations than the model resolution. Nonetheless, Figure 8d makes clear that most of the EPI in modern climate models is due to resolved precipitation, and thus it is to be expected that the EPE horizontal scale is mostly determined by resolved precipitation.

### 3.4 Role of Planetary‐Scale Dynamics

The analysis presented thus far shows evidence that both extreme ascent and EPE horizontal scales are resolution‐dependent in climate models, and the simulated EPE horizontal scales are determined by resolved (rather than parameterized) precipitation. There are several possible explanations for the latter result. One possible explanation is that the planetary‐scale dynamics, which can strongly influence EPEs in the extratropics, are also resolution‐dependent in the models.

**Figure 9:**
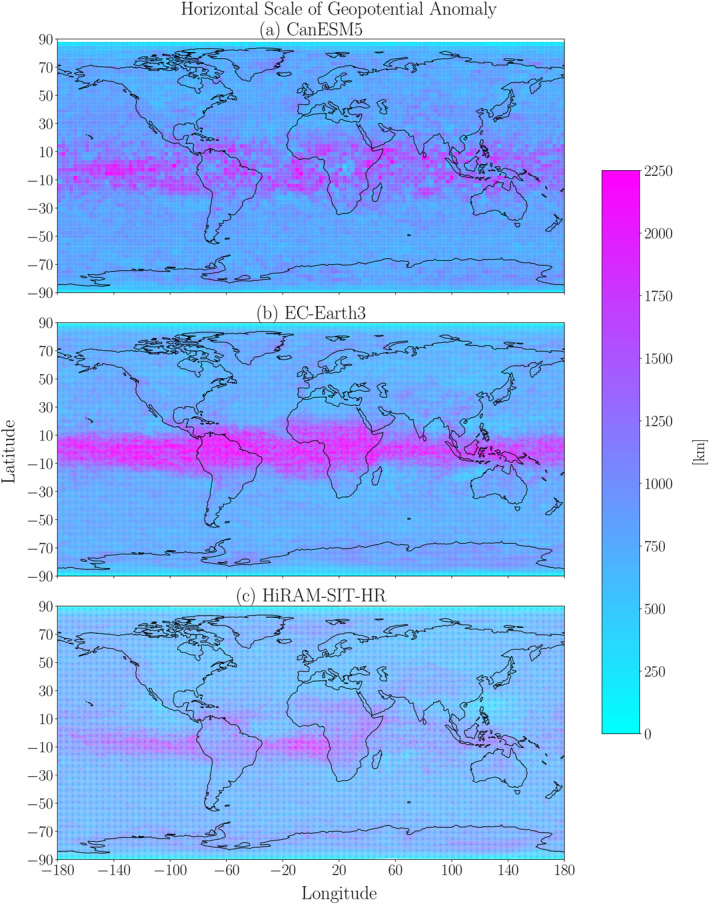
Global plots of the geopotential anomaly horizontal scales at 500 hPa averaged over EPEs during 1981–2000 for (a) CanESM5, (b) EC‐Earth3 and (c) HiRAM‐SIT‐HR.

Variations in geopotential are key indicators of variations in planetary‐scale dynamics, and the resolution dependence of such dynamics can be assessed by examining the horizontal scales of geopotential anomalies. Figure 9 shows global plots of geopotential horizontal scales for CanESM5, EC‐Earth3 and HiRAM‐SIT‐HR. The geopotential horizontal scales are maximum around the equator and lower elsewhere. This structure contrasts with those of the extreme ascent and EPE horizontal scales, which show more localized maxima over the midlatitude and subpolar regions (Figures 2 and 5).

Some regional variation of geopotential spatial scale in the extratropics becomes clearer when we adjust the shading scale to focus on the range of spatial scales in the extratropics (not shown). In particular, there are generally higher values over the subtropical oceans than elsewhere in the extratropics, in agreement with the results derived from reanalysis by Dai and Nie (2020) and from CMIP6 historical simulations by Dai and Nie (2022). CanESM5 shows stronger spatial variations of geopotential spatial scales compared to EC‐Earth3 and HiRAM‐SIT‐HR (not shown). Furthermore, these spatial variations appear to be more strongly correlated with vertical velocity spatial scales in CanESM5 than they are in EC‐Earth3 and HiRAM‐SIT‐HR. Thus, we expect (and we will confirm below) that the geopotential spatial scales will not show resolution dependence similar to that of the extreme ascent spatial scales.

Quantitatively, the geopotential horizontal scales are an order of magnitude larger than the extreme ascent and EPE horizontal scales, and they show a closer correspondence to theoretical expectations based on the Rossby radius of deformation, which is order 1,000 km. For example, the global mean geopotential horizontal scales for CanESM5, EC‐Earth3 and HiRAM‐SIT‐HR are around 880km, 900km and 798km, respectively. This contrast suggests that planetary‐scale dynamics is not the dominant process governing the EPE and extreme ascent horizontal scales. This contrast also sheds additional light on our earlier comparison with the results of Frierson et al. (2006) and Kidston et al. (2010). In particular, our quantitatively lower EPE and extreme ascent horizontal scales compared to those studies do not result from geopotential disturbances that shrink in size during EPEs. Rather, it appears that there are processes (such as grid‐scale convection) during simulated EPEs that appear to reduce their horizontal scale compared to the horizontal scale of geopotential disturbances.

**Figure 10:**
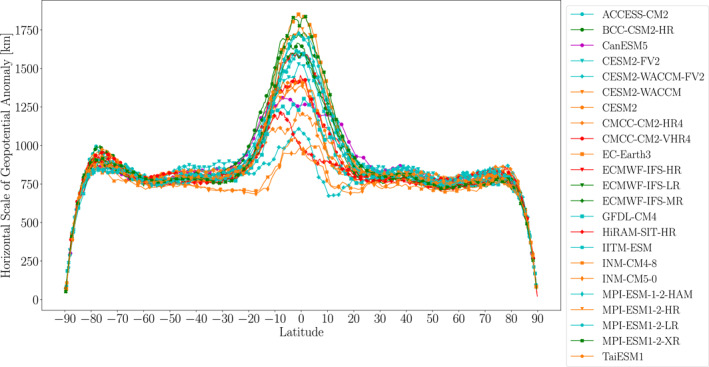
Zonal mean geopotential anomaly horizontal scales at 500 hPa averaged over EPEs during 1981–2000.

Figure 10 shows the zonal average of the geopotential horizontal scale for all models in this study. This calculation reiterates the clear maximum in horizontal scale in the tropics, which for some models is more than twice the horizontal scale in the extratropics. For most models, this peak occurs approximately on the equator, but for some models (e.g., HiRAM‐SIT‐HR) this peak is 10–15° south of the equator. As mentioned previously, the peak around the equator for the EPE and extreme ascent horizontal scales was not as prominent. The relatively large geopotential horizontal scales in the tropics makes the meridional variations in horizontal scale over the extratropics look much lower. Nonetheless, there are still modest peaks in horizontal scale in the midlatitudes and subpolar regions and sharp declines toward the poles, features also apparent in the EPE and extreme ascent horizontal scales. As was the case for extreme ascent and EPE horizontal scales, the intermodel spread is larger in the tropics than at higher latitudes. Over the extratropics, however, the intermodel spread in geopotential horizontal scales is low in relative terms (though similar in absolute terms) to the intermodel spread in EPE and extreme ascent horizontal scales. The geopotential horizontal scales shown here quantitatively agree with those shown in Dai and Nie (2020), Dai and Nie (2022).

**Figure 11:**
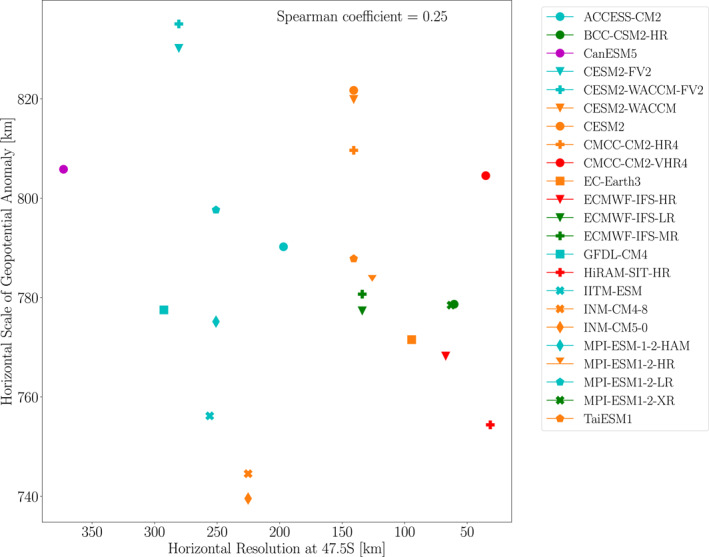
Geopotential anomaly length scales at 500 hPa averaged over EPEs during 1981–2000 and averaged over 55°S to 40°S latitude plotted versus horizontal model resolution at 47.5°S. The Spearman correlation coefficient is indicated in the upper right of the figure.

To assess possible resolution dependence, Figure 11 shows the geopotential horizontal scales averaged over 55–40°S plotted versus model horizontal resolution. Compared to the EPE and extreme ascent horizontal scales, the geopotential horizontal scales show no clear dependence on model resolution. Thus, the resolution dependence of EPE horizontal scales does not appear to be due to resolution dependence of planetary‐scale dynamics in the models.

Another possibility is that models are convecting on the model grid during EPEs, and this produces resolved precipitation with horizontal scale that is resolution dependent. We have performed analysis of the Canadian Earth System Model version 2 (CanESM2) which revealed that the timing of EPEs coincided with peaks in convective available potential energy (CAPE, not shown). This finding, along with the fact that the CanESM2 simulated precipitation during the EPEs is almost entirely resolved precipitation, supports the notion that the model is convecting on the model grid during EPEs. However, additional work is needed to examine this grid‐scale convection in more detail and assess its role in other models. If grid‐scale convection is the primary reason for the dominance of resolved precipitation during EPEs, that would be cause for concern since parameterized precipitation due to subgrid‐scale convection would be expected to play a more significant role during EPEs (Berg et al., 2013; Park & Min, 2017; Roca & Fiolleau, 2020). Another possibility that is worth exploring in future studies is whether there is formation of strong, poorly resolved horizontal pressure gradients that contribute to the strong resolved ascent and precipitation during simulated EPEs. Such a possibility is very plausible based on the model experiments and scaling analysis of Herrington and Reed (2018, 2020), which show that simulated pressure gradients (and hence vertical velocities) scale like the inverse of the model grid spacing.

### 3.5 Additional Sensitivity Tests

**Figure 12:**
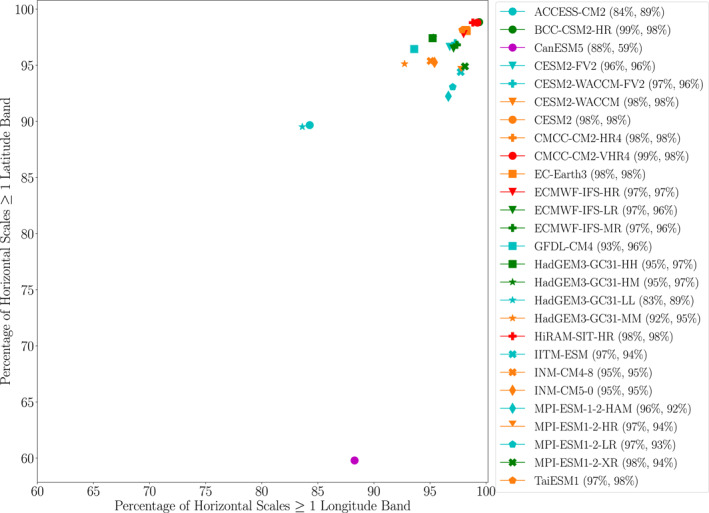
Percentage of EPEs for which the extreme ascent horizontal scale at 500 hPa in the (*x* axis) zonal and (*y* axis) meridional directions is greater than the grid spacing of the model. Percentages have been computed from all EPEs globally during 1981 to 2000. Each point corresponds to a calculation from a single model, and the coordinates for each point are also indicated in the legend.

There are a couple of additional possible explanations for the resolution dependence of EPE spatial scale that are worth addressing here. Firstly, if the EPEs in models were simply “grid box storms,” that is, strong anomalies confined to a single grid box, then one would expect there to be strong resolution dependence in the horizontal scale of EPEs. Our earlier analysis has already established that the extreme ascent and EPE horizontal scales are greater than the grid box size for all models when averaged over all EPEs. Figure 12 addresses this matter further by showing the percentage of EPEs globally for which the horizontal scale is greater than one model grid cell. This calculation reveals that, for all models, the majority (greater than 60%) of the EPEs have horizontal scales that are larger than a grid box in both the zonal and meridional dimensions. If the coarsest‐resolution model, CanESM5, is excluded, then over 80% of events in the models are greater than one grid box. These results confirm that the resolution dependence of simulated EPEs cannot be simply explained by grid box storms.

**Figure 13:**
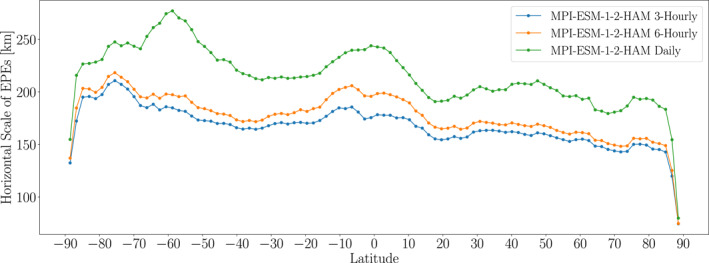
As in Figure 3a, but for EPE horizontal scales in MPI‐ESM‐1‐2‐HAM of (blue) maximum 3‐hourly, (orange) maximum 6‐hourly and (green) daily precipitation composited over the days of annual maximum precipitation during 1981–2000.

Another possibility is that, at time scales shorter than a day, the simulated EPEs are closer to grid box storms, and the precipitation system moves horizontally such that it appears to have a larger horizontal scale when averaged over a day. To explore this possibility, Figure 13 shows the EPE horizontal scales from MPI‐ESM1‐2‐HAM for the maximum of 6‐hourly (orange) and 3‐hourly precipitation (blue) on the day of annual maximum precipitation composited over 1981–2000. These computations reveal that, across all latitudes, the EPE horizontal scales decrease with the timescale of analysis. That is, EPE horizontal scales on 6‐hourly timescales are smaller than on daily timescales, and EPE horizontal scales on 3‐hourly timescales are smaller than on 6‐hourly timescales. The largest difference between the daily and 6‐hourly horizontal scales is approximately 80 km around 60°S, and the largest difference between the 6‐hourly and 3‐hourly spatial scales is approximately 30 km around the equator. These findings support the notion that advection is playing a role in increasing the EPE spatial scale on daily timescales compared to subdaily timescales.

If we disregard the suspiciously low polar values, the 3‐hourly spatial scales correspond to diagonal EPE sizes of 350 km or greater. (Recall from our discussion of Figure 4 that the diagonal size of an EPE is approximately 2.5 times the spatial scale.) For example, around 75°N the three‐hourly spatial scale is approximately 140 km, which translates to a diagonal size of approximately 350 km. This is larger than MPI‐ESM‐1‐2‐HAM's nominal resolution of 250 km as well as the model's actual resolution of 215 km at 75°latitude. However, given the closeness of the EPE size to the horizontal resolution, it cannot be ruled out that, at a given instant, the EPE is simply a grid box storm, and in future studies, additional analysis should be performed for multiple models to investigate this possibility more completely. In particular, grid scale convection that is sufficiently strong and localized could lead to grid box storms, and this possibility should be explored further in the future.

**Figure 14:**
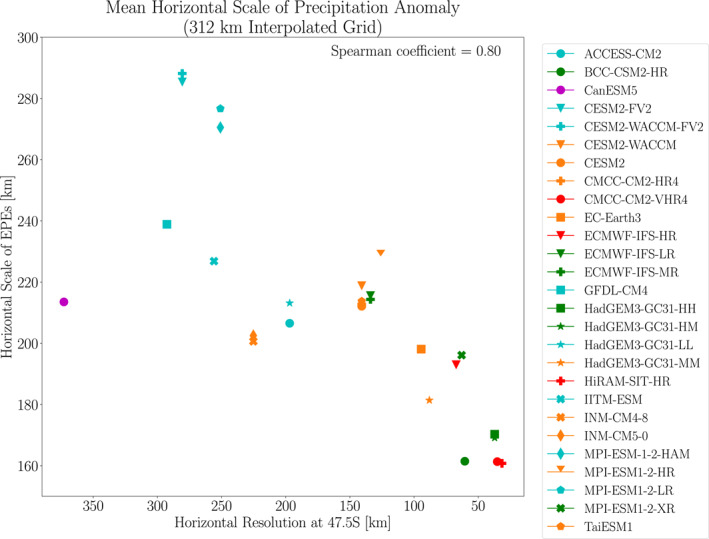
As in Figure 4a but for EPE horizontal scales computed after interpolating model output to a 312 km by 312 km grid (corresponding to the coarsest resolution model analyzed), rather than computing horizontal scales on the model's native grid.

A possible concern is that, by performing our analysis on the native grids of the models, we are automatically selecting for EPEs of different sizes, and thus it is expected that EPE horizontal scale increases with increasing grid spacing. On the other hand, if the timings of EPEs in neighboring grid cells sufficiently coincide with each other, then such an effect would not be significant. To assess possible sensitivity to our EPE selection approach, Figure 14 plots EPE spatial scale in the SH midlatitudes versus resolution after first interpolating the daily precipitation onto a 312 km by 312 km grid. (This grid box size corresponds to that of the coarsest resolution model, CanESM5, at the equator.) This calculation produces spatial scales that are overall about 20 km larger than those computed on the native model grids. But otherwise, the models show essentially the same resolution dependence as in Figure 4a, with an approximate 130 km drop in EPE horizontal scale over the model resolution range, and an intermodel correlation of 0.80. Thus, the resolution dependence of EPE horizontal scale is clear regardless of whether we perform our computations on a standard grid or on the native model grids.

## 4 Summary and Concluding Remarks

Our analysis of annual maximum of daily precipitation from CMIP6 output has shown that, in modern GCMs, the horizontal scales of EPEs and extreme ascent vary similarly in space. These spatial variations include local peaks over the subpolar regions and midlatitudes, with generally greater horizontal scales over the ocean than over land. This correspondence reaffirms the pivotal role of dynamics in the regional variation of EPE characteristics. However, there are large quantitative intermodel differences in horizontal scales, with approximately a factor of three difference in the midlatitudes and a factor of seven difference in the tropics between the models with the smallest and largest horizontal scales.

We have shown that horizontal model resolution is a key influence on this intermodel spread of EPE and extreme ascent horizontal scale. For a factor of seven increase in horizontal resolution, the EPE and extreme ascent horizontal scales decrease by a factor of approximately two to five, with higher sensitivity in the tropics than in the extratropics. Such resolution dependence is highly concerning because the extreme ascent horizontal scale influences the advective timescale and convective feedbacks, which in turn influence the accumulated precipitation (Dwyer & O’Gorman, 2017; Nie & Sobel, 2016; Tandon et al., 2018). Higher resolution models are generally better able to produce EPE horizontal scales in agreement with observational estimates, although in the extratropics, mid‐resolution models also produce horizontal scales that fall within the range of observational uncertainty.

Further investigation in the SH midlatitudes revealed that most of the precipitation during simulated EPEs is resolved rather than parameterized, and as a result, the simulated EPE horizontal scales are determined almost entirely by resolved precipitation. The planetary‐scale dynamics in the GCMs (as encapsulated by variations in geopotential) do not appear to be resolution dependent, so other possible explanations for the dominance of resolved precipitation need to be explored in future studies. These possibilities include convection on the model grid as well as formation of strong, poorly resolved fronts. Furthermore, much of our detailed analysis was performed over the SH midlatitudes, and additional analysis over other latitude bands is needed to be confident about the general applicability of our findings. As the required model output becomes available, assessing the resolution‐dependence of EPEs for a wider range of return periods and durations would also be valuable.

Clarifying the reasons why resolved precipitation dominates simulated EPEs will point to possible ways to improve GCMs and avoid resolution dependence in extreme precipitation simulations. Alongside these efforts, independent observational estimates of precipitation with adequate temporal and spatial resolution will need to be maintained in order to further assess the realism of GCM simulations of extreme precipitation.

## Data Availability

Details and DOIs for the observational products used in this study can be found in the references (specifically Xie et al., 2019; Huffman et al., 2019, 2022). CMIP6 model output can be obtained through the Earth System Grid Federation as described in Eyring et al. (2016). The codes used to analyze these data and produce the figures are available from the authors upon request.
